# Implementation of an online pain science education for chronic musculoskeletal pain in Brazilian public health system: protocol for a hybrid type III randomised controlled trial with economic evaluation

**DOI:** 10.1186/s12891-023-06360-7

**Published:** 2023-04-10

**Authors:** Marina P. Baroni, Luiz Hespanhol, Gisela C. Miyamoto, Christiane R. Daniel, Lívia G. Fernandes, Felipe J. J. dos Reis, Joshua W. Pate, Bruno T. Saragiotto

**Affiliations:** 1 Physical Therapy, Universidade Cidade de São Paulo (UNICID), São Paulo, SP Brazil; 2 Department of Physical Therapy, Universidade Estadual Do Centro-Oeste (UNICENTRO), Alameda Élio Antonio Dalla Vecchia, 838, CEP 85040-167, Vila Carli, Guarapuava, PR Brazil; 3 Centre for Pain, Health and Lifestyle, São Paulo, Brazil; 4 Amsterdam Collaboration On Health & Safety in Sports, Department of Public and Occupational Health, Amsterdam Movement Sciences, Amsterdam University Medical Centers (UMC) Location VU University Medical Center Amsterdam (VUmc), Amsterdam, the Netherlands; 5 Department of Health Science of Vrije, Universiteit Amsterdam, Amsterdam, the Netherlands; 6 Postgraduate Program in Medical Sciences, University of São Paulo (USP), São Paulo, SP Brazil; 7 Department of Physical Therapy, Instituto Federal Do Rio de Janeiro, Rio de Janeiro, RJ Brazil; 8 Discipline of Physiotherapy, Graduate School of Health, University of Technology Sydney, Sydney, Australia

**Keywords:** Chronic pain, Health education, Telehealth, Primary healthcare, Public health, Implementation science

## Abstract

**Background:**

Although clinical practice guidelines recommend pain education as the first-line option for the management of chronic musculoskeletal pain, there is a lack of pain education programmes in healthcare. Thus, digital health programmes can be an effective tool for implementing pain education strategies for public health. This trial will aim to analyse the implementation and effectiveness outcomes of three online pain science education strategies in the Brazilian public health system (SUS) for individuals with chronic musculoskeletal pain.

**Methods:**

We will conduct a hybrid type III effectiveness-implementation randomised controlled trial with economic evaluation. We will include adult individuals with chronic musculoskeletal pain, recruited from primary healthcare in the city of Guarapuava, Brazil. Individuals will be randomised to three implementation groups receiving a pain science education intervention (*EducaDo*r) but delivered in different modalities: group 1) synchronous online; group 2) asynchronous videos; and group 3) interactive e-book only. Implementation outcomes will include acceptability, appropriateness, feasibility, adoption, fidelity, penetration, sustainability, and costs. We will also assess effectiveness outcomes, such as pain, function, quality of life, sleep, self-efficacy, and adverse effects. Cost-effectiveness and cost-utility analyses will be conducted from the SUS and societal perspectives. The evaluations will be done at baseline, post-intervention (10 weeks), and 6 months.

**Discussion:**

This study will develop and implement a collaborative intervention model involving primary healthcare professionals, secondary-level healthcare providers, and patients to enhance self-management of chronic pain. In addition to promoting better pain management, this study will also contribute to the field of implementation science in public health by generating important insights and recommendations for future interventions.

**Trial registration:**

ClinicalTrials.gov (NCT05302180; 03/29/2022).

## Contributions to the literature

- Pain science education is effective for chronic musculoskeletal pain; however, there is a lack of specialised pain care in the public health system, especially in low- and middle-income countries.
- The literature is scarce on implementation trials testing innovative digital health solutions for healthcare.
- The implementation of the pain science education strategies in the Brazilian public health system (SUS) could provide information to discuss the best strategy and mode of delivery and will support the expansion of the implementation science in public health.

## Background

Chronic pain is commonly described as pain that lasts or recurs for longer than three months [1]. Most cases of chronic pain occur in the musculoskeletal system, such as osteoarthritis, back and neck pain [2]. The worldwide prevalence of chronic musculoskeletal pain is estimated at 30% in the adult population [3]. Musculoskeletal disorders are the top-ranked causes of years lived with disability (YLDs), accounting for 149 million of YLDs in 2019 globally [2]. The treatment of chronic musculoskeletal pain consists of reducing pain, maximizing function, and improving quality of life [4, 5]. Clinical guidelines commonly recommend non-pharmacological approaches as first-line management, such as exercises, manual therapies and pain education [6–9].

Pain science education provides knowledge and strategies for changing maladaptive beliefs and behaviours in face of pain, such as pain-related fear and avoidance [10, 11]. Pain science education, as one part of recommended multi-modal treatments, is effective in reducing pain [12, 13], anxiety, depression [13] and disability [14, 15], and in increasing knowledge about pain [15] in adults with chronic musculoskeletal pain. In addition to exercise-based treatment, pain science education is more effective in reducing pain (weighted mean differences: -2.09/10; 95%CI: -3.38 to -0.80) and disability (standardized mean difference: -0.68; 95%CI: -1.17 to -0.20), compared to exercise alone in the short-term [16]. Usual physiotherapy or exercise-based treatments alongside pain science education also can be cost-effective compared to usual care alone [17, 18].

Despite that, generally, evidence-based practice is inadequately integrated into lifestyle behaviours of individuals with chronic musculoskeletal pain [19–22]. Also, health policy and healthcare services deliver the treatments at a suboptimal level compared to the chronic musculoskeletal pain burden [19–22]. There is limited access to healthcare specialized in pain and health information [23], lack of skills among health workers for treating pain [5, 19, 24, 25], and limited options for biopsychosocial interventions in relation to the health system demand [26], and inadequate support for optimizing self-care [5, 24, 25]. In this context, the use of digital interventions to provide support for self-care management of health conditions attracts attention [27–29].

However, the literature is scarce in reporting the process and the outcomes of the implementation of interventions in digital models [20, 30]. Furthermore, evidence of chronic musculoskeletal pain clinical guidelines is derived largely from research on high-income economies [30]. Thus, Implementation research to bring the chronic musculoskeletal pain clinical guidelines into policy and ‘real-world’ practice in low- and middle-income countries is urgently needed.

The implementation of an online pain science education programme in Brazil can provide helpful information for low- and middle-income countries.

Therefore, the primary aim of this study is to investigate the implementation outcomes and effectiveness of three implementation strategies of an online pain science education programme in the Brazilian public health system of Guarapuava city. The second aim is to determine the cost-effectiveness of such strategies.

## Methods/ design

### Elaboration protocol

**Fig. 1:**
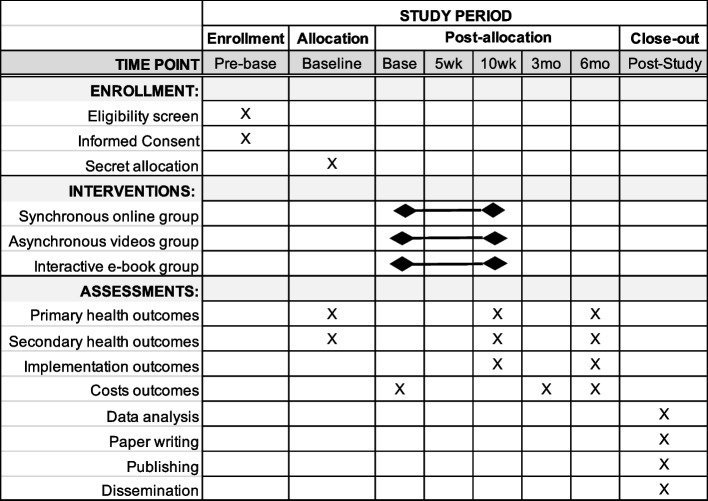
SPIRIT recommended content for the schedule of enrollment, interventions and assessments. Figure legend: wk: week; mo: month

This clinical trial protocol follows the recommendations of Standard Protocol Items: Recommendations for Interventional Trials (SPIRIT) [31] (Fig. 1), Consolidated Standards of Reporting Trials (CONSORT) [32], Consolidated Standards of Reporting Trials of Electronic and Mobile Health Applications and online TeleHealth (CONSORT-EHEALTH) [33], Template for Intervention Description and Replication (TIDieR) [34], and Consolidated Health Economic Evaluation Reporting Standards 2022 (CHEERS 2022) Statement [35]; and was planned using the implementation taxonomy proposed by Proctor (2011) [36].

### Trial design

This trial is designed as a hybrid type III effectiveness-implementation randomised controlled trial, including economic evaluation, with three arms [37].

### Ethical aspects

This study was revised and approved by the Research Ethics Committee of Universidade Estadual do Centro-Oeste (UNICENTRO/Brazil; CAAE 11,975,019.0.0000.0106; date: 07/15/2022). The protocol of this study was prospectively registered at ClinicalTrials.gov (NCT05302180; 03/29/2022).

### Brazilian Unified Health System (Sistema Único de Saúde – SUS)

The SUS is one of the largest public health systems in the world [38], covering the entire Brazilian population, and is used by about 75% of the population [39]. The SUS encompasses three levels of complexity: (1) primary healthcare (i.e., first contact, diagnosis and prevention); (2) secondary healthcare (i.e., specialised medical services, diagnostic and therapeutic support, and emergency services); and (3) tertiary healthcare (i.e., highly specialised care, surgery, cancer treatment and specific diagnosis procedures) [38, 40]. Primary healthcare is available in the entire country using the same system, but secondary and tertiary healthcare depends on each state and municipality, including the flow of users, triage processes, and organisation of the system [40].

### Guarapuava City, Parana State, Brazil

The municipality of Guarapuava is located in the Center-South region of the Paraná State, Brazil. Guarapuava has an estimated population of 183.755 inhabitants, distributed in 5 administrative districts (Entre Rios, Guairacá, Guará, Palmeirinha and Sede) [41, 42]. The city has a territorial area of 3,168.087 km^2^ (1.6% of the state of Paraná) with a population density of 53.68 inhabitants/km^2^. The Human Development Index (HDI) in 2010 was 0.731 [43] and a gross domestic product (GDP) per capita of R$ 33.914.00 in 2018 [44], similar to the Brazilian average in the same year (0.765 and R$ 32,747.00, respectively).

### Organisational settings of SUS in Guarapuava City

**Fig. 2:**
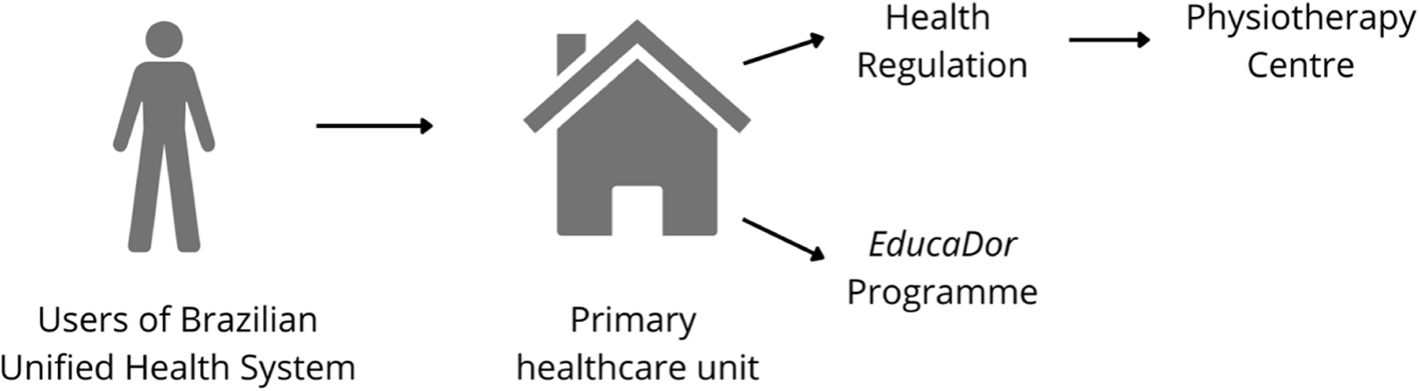
Schematic representation for the referral of SUS users with chronic musculoskeletal pain to *Educador* programme

Guarapuava has 33 primary healthcare units from SUS. Each primary healthcare unit has a defined population under its responsibility. When someone needs to use health services, the user is screened by a primary care professional, who determines the most appropriate level of healthcare attention the user needs. If the user needs a specialised health service (e.g., therapy) the health professional requests it to the public health regulation through the municipal electronic system. Thereafter, the public health regulation schedules the specialised care in the health units accredited by SUS and the user can receive the specialised care. There are five physiotherapy centres (secondary care) in Guarapuava. In this study, all patients scheduled at any of these physiotherapy centres will also be invited to participate in the online pain science education programme (named as *EducaDor*). The health professional of the primary healthcare unit can schedule the patient for the *EducaDor* programme through the municipal electronic system. A researcher from the *EducaDor* programme will assess the participant for eligibility criteria to participate in the study (Fig. 2).

### Settings and eligibility criteria

We will invite adults (18 years or older) with chronic musculoskeletal pain living in Guarapuava, Paraná State, Brazil, who were directed to physiotherapy based-care in SUS. The eligibility criteria for participating in this study are: (1) individuals who report chronic musculoskeletal pain (> three months); (2) those who speak in Portuguese; (3) those who have a smartphone, tablet, or computer with internet access; and (4) individuals who are undergoing or have been referred for physiotherapy based-care. Participants will be included in the study after agreeing to and accepting the online informed consent form.

### Procedures and randomisation

Individuals referred to the EducaDor programme will first be contacted by a researcher (MPB; BCG) via an electronic videoconferencing system at the scheduled consultation. During this initial contact, the researcher will screen the participant for eligibility and provide information about the EducaDor programme, as well as the name of the referred physiotherapy centre. Eligible participants will receive a link to an online consent form via email or text message, which they can accept to participate. Once the consent form is accepted, one researcher will conduct the baseline assessment, and another researcher will randomly assign the participant to one of the intervention groups.

Participants included in the study will receive physiotherapy-based care and the *EducaDor* programme as per randomisation into three different modes of delivery: (1) synchronous online group; (2) asynchronous videos group; or (3) interactive e-book group. Although the three groups have the interactive e-book, the synchronous online group and the asynchronous videos group receive additional types of digital content that are accessible to participants who cannot read and/or write in Portuguese. Then, the first step of randomisation will be done for participants who cannot read and/or write in Portuguese into (1) synchronous online group or (2) asynchronous videos group. The second step of randomisation will include all other eligible participants into the three different modes of delivery: (1) synchronous online group; (2) asynchronous videos group; or (3) interactive e-book group.

One researcher (ARF) will perform a stratified block randomization within the five physiotherapy centres accredited at SUS in the city, at the ratio of 1:1, using Excel software. The randomised list will be under the custody and confidentiality of the researcher (ARF). One week before the start of the *EducaDor* programme, one researcher (ARF) will send a list of participants to the researchers responsible for the interventions (MPB, KRM, MFG, and PAC). The intervention researchers will then contact the participants to explain the intervention according to group allocation.

### Data collection timepoints

We will evaluate participants at baseline, at the end of the intervention period (10 weeks), and 6 months after the randomisation. We will perform the evaluation through videoconference, telephone contact, or using an online questionnaire (e-mail or text message), according to the participant’s preference.

### Evaluation and data collection

#### Blinding

The outcome assessors will be blinded to the allocation of participants in the study groups. To assess the effectiveness of blinding, the assessors will be asked at the end of the study to guess which group they believe each participant was allocated to. However, due to the nature of the interventions, it will not be possible to blind the participants or the researchers responsible for delivering the interventions.

#### Characteristics of the sample

We will collect age, gender, body mass, height, marital status, profession/work, level of education, cognition [45], self-assessment of general health status, and drug and non-drug treatment [46].

#### Implementation outcomes

**Fig. 3:**
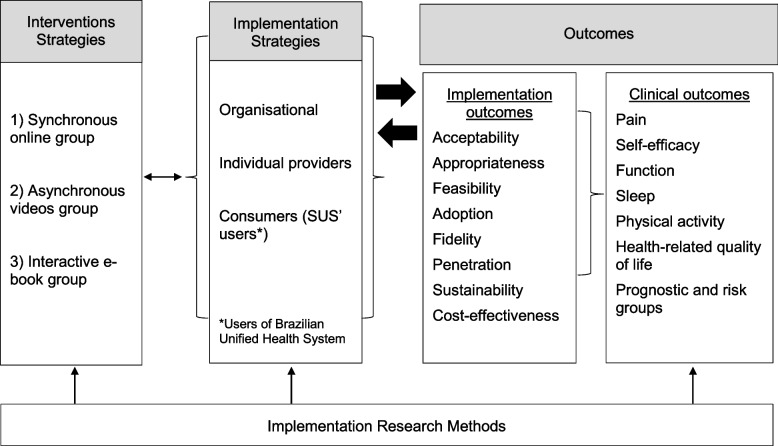
Adaptation of Proctor’s conceptual model of implementation research

Implementation outcomes will be used according to Proctor’s conceptual model of implementation research [36] (Fig. 3) and will be evaluated at different levels of analysis: (1) organisation (public health managers) and/or setting (health professionals, community health agents, primary healthcare unit’ leaders or other health servers involved in the implementation process); (2) end-users (participants from the SUS with chronic musculoskeletal pain); (3) individual providers (evaluators and intervention executing team).

The primary implementation outcomes will be cost-effectiveness, adoption, and acceptability (satisfaction using a numerical scale from 0 to 100). Primary and secondary implementation outcomes are described below and summarized in Table 1.

- *Acceptability* will be analysed from the perspective of the organisation through a focus group, from the perspective of end-users through an individual qualitative interview about satisfaction with the intervention, and by a numerical scale from 0 to 100 on how satisfied they were in participating in the intervention. The acceptability of the implementation will also be evaluated by the Acceptability of Intervention Measure (AIM) [47] in organisations and end-users. The AIM has four questions with a Likert scale from 1 (completely disagree) to 5 (completely agree), and the mean of the score will be used (1 to 5). Higher scores indicate greater acceptability [47].
- *Appropriateness* will be analysed from the end-users’ perspective regarding the perception of the appropriateness of each component of the *EducaDor* programme about their health condition through an adapted questionnaire [48]. This questionnaire will assess the degree to which participants agree with a series of statements about the intervention, on a Likert scale from 1 (totally disagree) to 4 (totally agree). The appropriateness of the implementation will also be evaluated by the Intervention Appropriateness Measure (IAM) [47] in the organisation and end-users. The IAM score is the same as the AIM.
  *Feasibility* will be evaluated from the perspective of the organisation and individual providers through a focus group, and the Feasibility of Intervention Measure (FIM) [47] will be used in participants, organisations and individual providers. The FIM score is the same as the AIM and AIM.
  *Adoption* will be analysed by the percentage of acceptance of public health users to participate in the *EducaDor* programme referred from primary healthcare. We will contact by telephone those public health users who refuse to participate by inviting them to an individual qualitative interview to identify barriers to engagement in the *EducaDor* programme.
- *Fidelity* will be evaluated by an independent pain specialist (FJJR), who will randomly select 10% of the recorded meetings and evaluate the intervention providers in relation to the fidelity of the intervention manuals with the content and quality of the meetings in a qualitative individual interview.
  The adherence of participants with the *EducaDor* programme will be measured by the frequency rate in synchronous meetings; self-rated performance of proposed homework on a numerical scale from 0 to 10 during the intervention period; and by exercise adherence scale (EARS-Br) [49].
- *Penetration* will be analysed descriptively from the rate of public health users referred from each primary healthcare unit.
- *Sustainability* will be evaluated by a focus group with public health managers after the presentation of the results of the study to discuss the maintenance of the *EducaDor* programme, and by a response rate of synchronous group users and reasons for discontinuity of the programme through an individual qualitative interview.
- *Costs* will be estimated from the SUS and societal perspectives in a time horizon of 6 months. The SUS costs will include the intervention costs and healthcare utilization costs covered by SUS. The intervention costs will be determined by preparation, video recording, development and editing, maintenance and support technology costs, monitoring data costs and staff, training health professionals and project management costs, number and duration of phone calls and the number of text messages sent to participants. The e-book and asynchronous intervention costs will be diluted in 10 years. The healthcare utilization costs covered by SUS will include prescribed drugs, medical consultations, physiotherapy-based care, visits to specialists, diagnostic exams, emergency services and hospitalisation. The societal costs will include the SUS costs, private healthcare costs, patient costs (out-of-pocket expenses with unprescribed drugs, complementary costs, and transportation costs), and lost productivity costs (related to absenteeism). The quantities of resource utilization will be identified by the participants in a self-rated diary cost that will be provided at baseline and an assessor will collect this information by telephone every 3 months. The healthcare utilization costs covered by SUS will be valued using the Brazilian standard costs [50]. Patient costs will be valued using the out-of-pocket expenses reported by patients. Private transportation will be valued by the price of Brazilian gasoline per kilometre and public transportation by the reference price of Guarapuava city. The lost productivity costs will be estimated from the hours of absenteeism to work (paid and unpaid) through a questionnaire, and evaluated according to the Human Capital Approach and will be valued using gender-specific price weights [50].

**Table 1:** Implementation outcomes

**Outcomes**	**Level of analysis**	**Indicators**		**Data Collection Tool**		**Time of data collection**
**Acceptability**	Organisation	Acceptability of the implementation		Qualitative interview and Acceptability of Intervention Measure (AIM)		Post-implementation
	Participants	Satisfaction with the intervention		Qualitative interview and 0 to 100 points scale and AIM		Post-implementation
**Appropriateness**	Participants	Are the components of the online pain science education programme appropriate for the health condition?		Qualitative interview, Intervention Appropriateness Measure (IAM) and an adapted questionnaire used by Liao et al. (2020)		Post-implementation
	Organisation	If intervention is appropriate for the public health condition		Intervention Appropriateness Measure (IAM)		Post-implementation
**Feasibility**	Participants	Feasibility of the intervention		Feasibility of Intervention Measure (FIM)		Post-implementation
	Organisation	Feasibility of the implementation		Qualitative interview and FIM		Post-implementation
	Individual providers	Feasibility of the implementation		Qualitative interview and FIM		Post-implementation
**Adoption**	Participants	Enrolled participants being contacted		Own domain database		During implementation
		Burden (reasons for not taking part)		Qualitative interview during triage		Pre-baseline
**Fidelity**	Creator of the intervention	10% of taped sessions of the intervention group were randomly selected and reviewed by an expert panel, against the full detailed intervention manuals for adherence and quality		Qualitative interview		Post-implementation
	Participants	Number of participants that adhered to the intervention		Frequency during intervention		During implementation
		Self-rated performing the proposed activities at home for 10 weeks and the 6-month of follow-up		Adherence to the proposed homework (0 to 10 points scale); and the exercise adherence scale (EARS-Br)		Post-implementation
**Penetration**	Organisation	Which primary healthcare units referred users with musculoskeletal pain for intervention?		Health regulation data and/or digital health record		During and post-implementation
**Sustainability**	Organisation	Do you think this project will be sustainable over time?		Qualitative interview		Post-implementation
	Participants	Indicator of drop in the response rate of the synchronous group		Own domain database		During implementation
		Burden (reasons for discontinuation or dropping out)		Qualitative interview		During implementation
**Costs**	Participants	Self-rated costs for the treatment of chronic pain		Diary of cost		During and post-implementation
	Organisation	Costs of public healthcare		Domain database and health regulation data		Post-implementation
	Individual providers	Costs of the intervention		Survey		Post-implementation
	Researchers team	Cost-effectiveness		Economic evaluation (incremental cost-effectiveness ratio – ICER—of the three strategies of the intervention)		Post-implementation
		Cost-utility		Economic evaluation (measured by the EQ-5D-3L to calculate quality-of-life-adjusted life years)		Post-implementation

#### Effectiveness outcomes

Health outcomes will be evaluated at baseline, at the end of the interventions (10 weeks) and 6-month follow-up. The primary effectiveness outcome will be current pain intensity, assessed in a one-dimensional aspect by the numerical rating scale (NRS) of 11 points, from 0 (no pain) to 10 points (the worst possible pain) [46]. The secondary effectiveness outcomes will be:

- *Pain:* the multidimensional aspect of pain will be evaluated by the Brief Pain Inventory [46]. The Brief Pain Inventory is a self-rated instrument and allows pain assessment in two dimensions: (1) pain intensity (items 3 to 6 of inventory); and (2) pain’s interference in the participant’s life (items 9a to 9 g of inventory). Each question has an NRS of 11 points, ranging from 0 (no pain/ no interference) to 10 (worst pain/ worst interference) and the average is used for each dimension.
- *Self-efficacy:* evaluated through the Chronic Pain Self-Efficacy Scale (CPSS) [51], with 22 items of individual’s beliefs, whose items are divided into three factors: (1) self-efficacy for pain control (AED); (2) self-efficacy for other symptoms (AES); and (3) self-efficacy for physical function (AEF). It is possible to obtain a score for each factor (score from 10 to 100), and the sum of all factors that range from 30 to 300 points. Higher scores demonstrate a greater individual’s ability to deal with the consequences of pain [51].
- *Function:* The Patient-Specific Functional Scale (PSFS-Br) will be used. The participant chooses 3 to 5 important activities in which they have greater difficulty due to their condition and then graduates the level of their difficulty on an 11 points scale, from 0 (inability to perform the activity) to 10 (capable of performing the activity at the same level as before the injury or problem) [52].
- *Sleep quality* will be evaluated by a self-rated sleep quality in the last 7 days on a scale of 0 to 100 points (0 – worst sleep quality; 100 – better sleep quality).
- *Health-related quality of life* will be evaluated using the EQ-5D-3L questionnaire, which is composed of a descriptive model with five health domains (mobility, self-care, usual activities, pain/discomfort, and anxiety/depression). Each domain has three response levels: ‘no problems’, ‘some problems’, and ‘extreme problems’. The instrument also has a visual analogue scale (EQ-VAS) for the self-rated health state which ranges from 0 (‘the worst possible health status) to 100 (‘the best possible health status’) [53, 54].
- *Prognostic stratification:* will be evaluated by the Keele STarT MSK Tool [55]. The tool contains 10 items (ranging from 0 to 12 points each) that, once scored, can place patients into three categories based on their risk of a poor outcome: (1) low risk (0–4 points); (2) medium risk (5–8 points); or (3) high risk (9–12 points).
- *Adverse events:* will be analysed by recording the number and type of adverse effects that occurred during the intervention period.

### The online pain science education programme (EducaDor)

The online pain science education programme (*EducaDor*) will be based on the model proposed by Reis et al. [56, 57]. The *EducaDor* programme will follow 10 steps that will be divided into videoconferencing meetings, videos, and e-book during the study: (1) acceptance; (2 and 3) pain education; (4) sleep hygiene; (5) pharmacological assistance; (6) recognizing stress and negative emotions; (7) increasing positive coping in lifestyle; (8) exercises; (9) communication; and (10) prevention of recurrence [56]. The content of these 10 pain science education steps also was written into an interactive e-book with simple writing and based on scientific literature.

Each chapter of the interactive e-book features a rich learning environment (texts, images, graphic schemes, podcasts, videos, and behavioural strategy activity) to provide a multisensory-training protocol and to produce greater and more efficient learning [58, 59]. Each chapter also contains behaviour strategies and activities for participants to develop during the week, to facilitate the comprehension of which changes that can be made in their daily life to have an impact on self-management and self-control of pain. All participants will receive the interactive e-book on their mobile phone, tablet or computer. It is expected that participants read and practice the activities and behavioural strategies, at least, one chapter per week.

- ***1) Synchronous online group***

Participants will receive the interactive e-book of the *EducaDor* programme on their smartphone devices and e-mail at the beginning of the programme, and 10 synchronous online groups (one per week), in addition to physiotherapy-based care.

The synchronous online *EducaDor* programme will be held in groups of up to 12 participants, during 10 weekly synchronous meetings on the Whereby® platform on a date and time pre-established by the research group, which may be changed according to participants’ preferences. The meeting link will be available by text message and e-mail few minutes before the meeting time.

In the first meeting, we will also promote a conversation with the group to know about the health conditions of each participant, and their expectations with the programme and explore previous experiences regarding treatments received, phobias and beliefs about pain, injury and interventions. The professional will conduct each synchronous meeting with dialogued exhibition class, that is, there will be a presentation using multimedia material [57] shared on the computer screen and thus will be accessible for participant viewing on his computer, tablet, or smartphone during the meeting. At the same time, there will be an explanation about the topic of the meeting with the health professional. The professional will conduct the meetings using clear, objective, and assertive communication, aiming to promote reflection and behaviour change in the participants’ daily life. After the topic exhibition, participants will be encouraged to participate by exposing their doubts and/or sharing experiences.

Finally, the professional will accomplish the activity proposed in the respective chapter of the interactive e-book, and will make orientations for performing during the week.

- ***2) Asynchronous videos group***

Participants allocated in the asynchronous videos group will receive the interactive e-book at the beginning of the programme and 10 videos (one per week) with the same topics of the synchronous online *EducaDor* programme sent on their smartphone devices and e-mail, in addition to physiotherapy-based care. Before receiving the materials, users will participate in an individual or group synchronous meeting of up to 12 participants on the Whereby® platform to receive a session of pain education, guidance for the use of the interactive e-book, and access to the videos over the 10 weeks. The videos were previously developed and tested in another clinical trial [60].

- ***3) Interactive e-book group***

Participants allocated to this group will receive the interactive e-book of the *EducaDor* programme on their smartphone devices and e-mail, in addition to physiotherapy-based care. Before receiving the e-book, the users will also participate in an individual or group synchronous meeting of up to 12 participants on the Whereby® platform to receive a session of pain education, and guidance for the use of the interactive e-book over the 10 weeks.

#### Strategies for engagement

All groups will receive weekly text messages encouraging participants to carry out the weekly homework available in the interactive e-book and/or videos. Participants will be asked about the difficulties in carrying out the homework and instructed on how to overcome them.

#### Fidelity of intervention delivery

The interventions will be conducted by four physiotherapy students (FCO, KRM, MFG, and PAC) from Universidade Estadual do Centro-Oeste (UNICENTRO/Brazil). The team will receive 30 h of training regarding the interventions before the beginning of the *EducaDor* programme. The chief researcher of the study (MPB), who has 18 years of physiotherapy experience, will audit the online interventions once every month. To maintain the fidelity of the intervention delivery, the researchers will follow a structured manual of the *EducaDor* programme. All synchronous meetings will be recorded for further evaluation of the implementation fidelity.

#### Physiotherapy-based care

All participants will receive physiotherapy-based care in one of the five physiotherapy centres accredited to SUS, according to the scheduling availability of the services. The physiotherapy-based care consists of 10 sessions, mainly based on exercises and electrophysical agents. Patients will be treated in an outpatient physiotherapy centre by independent therapists to minimize the possible preference bias of therapists.

### Implementation strategies

The implementation strategies of the *EducaDor* programme have been developed alongside municipal health managers. Potentialities, barriers and solutions to promote the implementation of the *EducaDor* programme were discussed with health managers. Other ones were discussed with the research group to reorganise the operationalisation of the study phases (Table 2).

**Table 2:** Potentialities, barriers and solutions to promote the implementation of the *EducaDor* programme

**Implementation domains**	Facilitators	Barriers	Solutions
**Intervention characteristics**	Stakeholders' perceptions of the quality and validity of evidence support the belief that the intervention will have desired outcomes and perception of the advantage of implementing the intervention and its adaptability to meet local needs The interventions can be tested and can reverse the implementation if warranted	(1) Difficulty of implementation in primary healthcare (2) Costs of the intervention and costs associated with implementation	(1) Implementation of interventions in the secondary level of healthcare (2) Low-cost interventions, PPSUS resources, and implementation of interventions in partnership with universities^a^ through research and extension projects
**Outer setting**	The public health organisation recognizes the high prevalence of chronic pain among SUS users by the number of requests for physiotherapy based-care for chronic pain treatment regulated by the municipality Also recognizes the evidence about the use of pain science education in chronic pain treatment, but this intervention is not yet available for SUS’s users in the municipality With the COVID-19 pandemic, the public health organisation of Guarapuava city could identify that most SUS users have smartphones and the internet, which can become these online interventions possible to occur There is a high network between public health organisations and the University (intervention providers)	(1) Currently, the public health organisation of Guarapuava city is not able to offer pain science education for SUS users in primary healthcare (2) Although the SUS’s users have access to smartphones and the internet, the stakeholders identified that the SUS’s users change their telephone numbers frequently (3) There is no pay-for-performance	(1) Implementation of the intervention in partnership with Unicentro, through the Clinical School of Physiotherapy, which is accredited to the secondary level of healthcare (2) Explain the importance of maintaining the telephone number over time; request home visits from community health agents to update the register (3) For the maintenance of the intervention after the study, we will request an increase in the number of service quotas to the Clinical School of Physiotherapy from the Health Department of the State of Paraná
**Inner setting**	The stakeholders agree that the intervention proposed is aligned with the norms, values and perceived risks and needs of the health policies They shared the perception of the importance of implementing the intervention and an online programme fit with the existing workflows and system in the municipality's health department considering the social distance imposed by the COVID-19 pandemic and the in-person return of other repressed care during the period of the pandemic Formal communication within the organisation for the referral of SUS’s users from primary healthcare to specialized healthcare (that is, to the secondary level of healthcare) is done through the information and technology system acquired by the municipal health department	(1) How to refer SUS users with chronic pain from primary healthcare to the *EducaDor* Programme (2) Health professionals' lack of knowledge about the new intervention in the digital information and communication system	(1) Provide referral access to the intervention in the digital information and communication system (2) Create a highlight on the intervention icon in the digital information and communication system, and insert video and explanatory text about the *EducaDor* programme; disseminate the video and text on the smartphones of health professionals in primary healthcare
**Characteristics of individuals**	The municipal health department organized study groups in various health thematic areas (eg, chronic diseases, elderly health, women's health, child and adolescent health, and others). Each study group is coordinated by a case manager, who organizes meetings with health agents, health professionals and municipal managers to discuss health cases and promote lectures to encourage continuing education. The study groups share about the health process in the respective primary healthcare unit, challenges and achievements. The case manager assists the team's engagement in strategic actions of municipal public health and represents a formal influence in the organisation on the attitudes and beliefs of colleagues about the interventions proposed for implementation. This relationship between public servants and managers can facilitate the relationship between them and SUS users, increasing the degree of commitment to public health	(1) Non-reference of SUS users with chronic pain to *EducaDor* programme due to lack of knowledge of health professionals about evidence-based practice	(1) Understand how evidence-based practice in primary healthcare is developed, specifically referring to *EducaDor* programme; explaining about pain science education and our programme to each case manager; participate in the study groups with the focus on chronic pain; disseminate the intervention to each primary healthcare unit leader
**Process of implementation**	Case managers and their study groups could facilitate the dissemination of adequate information at the various organisational levels of municipal public health and can assist in the process of implementing the *EducaDor* programme	(1) Lack of knowledge about pain science education on the part of physiotherapists who will be conducting physiotherapy-based care may discourage users from continuing the intervention	(1) Explain pain science education and our programme in-person to each physiotherapy centre accredited to the secondary level of healthcare

*SUS* Brazilian Unified Health System, *PPSUS* Research programme for SUS

^a^Universidade Estadual do Centro-Oeste (UNICENTRO): responsible for carrying out the interventions; e Universidade Cidade de São Paulo (UNICID): monitoring and guidance for the implementation process

The implementation process will be monthly monitored and evaluated on barriers and facilitators allowing adaptations throughout the implementation process of the *EducaDor* programme.

### Data management and statistical analysis

#### Data management

All data and materials will be used only for analysis of the present study and will be protected from any unnecessary exposure. The informed consent form will be digitally authenticated by researchers and patients. The recorded synchronous online meetings and audio from qualitative interviews will be available to the researchers only, and stored following the Brazilian General Law for Protection of Personal Data (LGPD). All data will be available for review and confirmation of data analysis when requested in the review process of publication without identifying the participants.

#### Sample size estimation

The sample size was estimated by simulation [61, 62]. The sample size simulation was performed in four steps: (1) defining the sample size problem and outcomes; (2) defining, writing and running the sample size simulation algorithm; (3) estimating the possible outcomes derived from the simulations; (4) finding the optimal sample size.

In step 1, the sample size problem was developed considering the following objective: to estimate the minimum sample size required to comply with a maximal type I error (*α*) of 0.05 or 5% and a maximal type II error (*β*) of 0.20 or 20%. The input outcomes were: populational mean pain intensity (0–10) of 6.0 with a standard deviation (SD) of 2.3; a hypothesised minimal clinically important difference (MCID) in pain intensity between groups of -1.0 with an SD of 0.25 [63]; three groups; three repeated measurements (baseline, 10 weeks and 6 months); a correlation within the repeated measurements of 0.30 or 30% [64]; a minimum of 30 participants per cluster (five physiotherapy facilities); a correlation within clusters of 0.20 or 20% [65]; and a loss to follow-up of 30% [66].

In step 2, the simulation algorithm was defined and written in R language [67]. The input outcomes defined in step 1 were then included in the algorithm. Normal distributions were simulated using the input means and SDs set in step 1 as hyperparameters. The simulations were gathered running the distributions considering several possible sample sizes for one group ranging from 5 to 5000 in 32 steps (i.e., 5 to 50 by 5; 60 to 100 by 10; 150 to 500 by 50; 600 to 1000 by 100; and 2000 to 5000 by 1000), with 100 replications for each step, and repeating this procedure 10 times (i.e., 10 iterations), summing up 32,000 sample size simulations in total.

In step 3, we estimated summary statistics for the simulated distributions in each of the 32 possible sample sizes, including the probability of type I error (*α*), type II error (*β*) and power (1-*β*). In step 4 we found the minimum sample size required to achieve the pre-defined type I error (*α* ≤ 0.05 or 5%), type II error (*β* ≤ 0.20 or 20%) and power (≥ 0.80 or 80%). At this moment the suggested minimum sample size was 80 participants per group. Then, corrections for loss to follow-up and correlations amongst the five clusters (physiotherapy facilities) and repeated measurements were applied [64, 65]. The sample size simulation suggested a minimum sample size of 105 participants per group, that is, 315 participants (105 in each treatment group) in total for this study.

#### Missing data

An intention-to-treat approach (ITT) will be used in the main statistical analyses, which will include all randomized participants. The ITT analysis aims to estimate the population’s average causal effect by considering the randomised allocation regardless of whether the participants in each group complied or not with their allocation condition [68]. A complier average causal effect (CACE) will also be used to estimate the local average causal effect within the compliers [68]. ‘Compliers’ will be considered those participants who pointed at least 17 points in the adherence questionnaire (EARS-Br) [49].

#### Quantitative data analysis

For continuous variables, we will calculate statistics of central tendency and dispersion, such as means and, standard deviation. For categorical variables, we will describe frequencies and absolute numbers. Normality will be investigated by visual inspection of histograms. The baseline characteristics of the participants and implementation outcomes will be calculated using descriptive statistics. The differences between groups and the 95% confidence interval will be calculated using mixed models. The fixed effect term will be composed of dummy variables indicating groups, and follow-up time-points, that is, 10 weeks and 6 months after baseline, and the interaction terms composed of group and time. The estimates will be adjusted for any potential differences that might exist at the baseline. The random effect term will be composed of: (1) a correlated random intercept and slope varying the intercept for the five physiotherapy clusters and varying the slope for time points; and (2) a correlated random intercept and slope varying the intercept for repeated measurements within each cluster and varying the slope for time-points [69].

#### Economic evaluation

The economic evaluation will be conducted with an ITT approach. The cost-effectiveness analysis will be conducted using pain (measured by the numerical pain scale after 6 months of randomization). The cost-utility analysis will be conducted using quality-adjusted life-years (QALYs). The QALYs will be estimated from the evaluation of the health status of the participants using the EQ-5D-3L instrument. These health states of the descriptive system of the EQ-5D-3L will be converted into utility values using Brazilian tariffs [54]. Finally, the QALYs will be calculated from the linear interpolation between measurement points using the utility values of the participants collected at baseline, 10 weeks, and 6 months after randomization.

The average cost differences between groups will be calculated for SUS and societal perspectives and disaggregated by cost categories. The average cost and effect differences will be estimated by unrelated regression analyses. The incremental cost-effectiveness ratios will be calculated by the difference between the costs of the interventions divided by the difference between the effects of these interventions. Bias-corrected and accelerated bootstrapping techniques (5000 replications) will be performed to estimate the uncertainty surrounding cost difference and incremental cost-effectiveness ratios. The cost-effectiveness pairs from the 5000 replications will be presented graphically in a cost-effectiveness plane. Cost-effectiveness acceptability curves will be estimated to indicate the probability of interventions being cost-effective compared to each other at different willingness-to-pay thresholds [70].

Multiple imputations by chained equations will be used to handle the missing cost and effect data. An imputation model will be developed and will include sociodemographic and anthropometric variables, duration of symptoms and all available values of costs and effects measured at baseline and follow-up periods. Ten complete datasets will be created (considering loss of efficiency < 5%). The grouped estimates will be calculated according to Rubin’s rules [71].

To evaluate the robustness of the results, two exploratory sensitivity analyses will be performed. The first sensitivity analysis will be performed by excluding the total costs of the e-book and the asynchronous video development and editing. The second sensitivity analysis will be performed considering only patients with more than 75% of adoption of the intervention. The economic evaluation will be conducted at RStudio.

#### Qualitative data collection

We will perform a focus group with the organisation and individual providers after the end of the *EducaDor* programme implementation. The interview will be in person or by videoconference using the Whereby® platform. The individual qualitative interviews will be done with a random sample of the end-users by videoconference or telephone call after the end of the intervention period.

Focus group and individual qualitative will be conducted by the same researcher and they will be recorded and subsequently transcribed *verbatim.* The interviews will occur in a semi-structured format and the interviewer will be able to organise the questions in the way that seems most comfortable, making use of specific techniques (*probing*) to deepen the topics brought by the participants. The same researcher will be responsible for the transcription of the recorded material, and another researcher will evaluate a sample of transcribed material with their respective audios to observe its accuracy and fidelity. The transcripts will be carried out simultaneously with the interviews, and the sample size will be determined using the concept of saturation [72]. Specifically, data collection will continue until the point where no new information emerges from the interviews regarding implementation outcomes based on a theoretical model of saturation [73]. At this stage, data collection will be deemed complete.

#### Qualitative data analysis

The qualitative analysis will consist of an iterative approach of thematic content analysis (*phronetic analysis*) described by Tracy (2007) [74]. The process will consist of four phases: (1) organisation and preparation of the data, with consequent cleaning of the data (where there was a clipping of the content); (2) line-by-line coding aiming to identify words or small phrases that could descriptively synthesise the content brought; (3) creation of a codebook with a list of identified codes bringing a small explanation, definition, or example of illustration; (4) second round of coding to revisit the codes presented in the codebook, organisation and categorisation in a more interpretative and analytical way. Constant comparations in phases I-IV will be used. Two authors will conduct phases I, II and III independently. 30% of the transcribed material from the first block of participants will be analysed for both authors’ agreement on the codebook. This codebook will guide the thematic analysis of the remaining interviews. In phase IV, both authors will meet again to achieve agreement on the topics raised. In case of disagreement, a third author will be consulted. In the end, all researchers will be consulted to confirm if the topics and subtopics reflect the primary data from the interviews.

#### Secondary analysis

Secondary studies will be described aiming:

1. Subgroup analysis to compare the effectiveness outcomes between the three intervention groups according to the participants’ baseline prognostic and risk groups [75]. Subgroups will be each prognostic and risk category (low, medium and high risk). A test for interaction between the intervention group and each subgroup variable (i.e., baseline prognostic and risk groups) will be performed to assess whether the intervention effect varies across the subgroups. For each subgroup, the mean difference will be estimated using linear regression. The results of the subgroup analysis will be reported in the trial manuscript or a separate publication.
2. An individual qualitative interview with end-users to identify the barriers and facilitators of the proposed interventions.

## Discussion

This study will develop and implement a collaborative intervention model involving primary healthcare professionals, secondary-level healthcare providers, and patients to enhance self-management of chronic pain. In addition to promoting better pain management, this study will also contribute to the field of implementation science in public health by generating important insights and recommendations for future interventions. We hypothesize that there will be different costs between the three modalities of the *EducaDor* programme, with the synchronous online group having a lower cost. Although the scalability and sustainability of the synchronous online group seem to be lower than the asynchronous modalities. We also hypothesize that the *EducaDor* programme will be implementable for patients with chronic musculoskeletal pain in the Brazilian public health system, and the synchronous online group and asynchronous video group will be the most cost-effective modalities. This study will provide information for further discussion with public health managers regarding the feasibility and sustainability of the *EducaDor* programme.

Although pain science education is important to be implemented in the healthcare system, knowledge about pain science related to pain conditions in Brazil is recent and still needs to be implemented in most academic curricula of health graduations, including physical therapy [24, 25]. Only 26 (6.5%) of those physical therapist education programmes available curricula on the website had a specific course about pain, covering a mean of 44.3 h [25]. It is unknown whether pain content is embedded in the curriculum throughout different subjects, rather than an entire subject about pain. Thus, not all undergraduates and physiotherapists have specific education to apply pain science education in addition to physiotherapy-based care [25]. The contemporary approach to pain science requires patient-centred care, and includes the understanding of different interactions between physical and mental dysfunctions and promoting self-management of health. An online pain science education could allow greater access to the population [76].

It must also be considered that the digital modality of physiotherapy-based care is recent in Brazil, being authorised as an alternative to healthcare during the COVID-19 pandemic. The pandemic itself intensified the health disparities of individuals suffering from chronic musculoskeletal pain [77]. In this context, digital health strategies emerge as an innovative option that associated with public and social health policies can provide greater access and contribute to reducing inequity of health access [78].

It is known that digital pain science education programmes provide improvement in health outcomes and benefits from self-management strategies including the return to activities, physical activity practice, behavioural changes and adequate knowledge about pain in people who suffer from chronic musculoskeletal pain [12, 79]. However, the results of the implementation of this service in the secondary level of healthcare are still unknown. Thus, it is necessary to analyse the implementation outcomes by users and managers in public healthcare to identify the best way of delivering content on pain science education [79].

The *EducaDor* programme was designed to enable the delivery of content synchronously and asynchronously, with simple, clear and direct language, on easy-to-handle platforms, with some human support to stimulate engagement in chronic musculoskeletal pain self-management interventions. Thus, the results of our study can contribute to the discussion of the implementation of online pain science education programmes in the specialised level of care for public health users, coordinate decision-making by primary healthcare professionals and foment the expansion of pain service in public health.

## Data Availability

All data and materials will be used only for analysis of the present study and will be protected from any unnecessary exposure. The informed consent form will be digitally authenticated by researchers and patients. The recorded synchronous online meetings and audio from qualitative interviews will be available to the researchers only, and stored following the Brazilian General Law for Protection of Personal Data (LGPD). All data will be available for review and confirmation of data analysis when requested in the review process of publication without identifying the participants.

## Abbreviations

YLDs: Years lived with disability
SUS: Brazilian Unified Health System
PPSUS: Research programme for SUS
VAS: Visual analog scale
AIM: Acceptability of Intervention Measure
IAM: Intervention Appropriateness Measure
FIM: Feasibility of Intervention Measure
UNICENTRO: Universidade Estadual do Centro-Oeste
UNICID: Universidade Cidade de São Paulo
